# 2-[*N*-(2,4-Dimeth­oxy­phen­yl)acetamido]-1,3-thia­zol-4-yl acetate

**DOI:** 10.1107/S1600536813003474

**Published:** 2013-02-09

**Authors:** Volodymyr Horishny, Roman Lesyk, Marcin Kowiel, Andrzej K. Gzella

**Affiliations:** a Department of Pharmaceutical, Organic and Bioorganic Chemistry, Danylo Halytsky Lviv National Medical University, Pekarska 69, Lviv, 79010, Ukraine; b Department of Organic Chemistry, Poznan University of Medical Sciences, ul. Grunwaldzka 6, 60-780 Poznań, Poland; c Faculty of Pharmacy, Ludwik Rydygier Collegium Medicum in Bydgoszcz, Nicolaus Copernicus University in Torun, ul. A. Jurasza 2, 85-089 Bydgoszcz, Poland

## Abstract

The title compound, C_15_H_16_N_2_O_5_S, is a product of the reaction of 2-(2,4-dimeth­oxy­phenyl­amino)-1,3-thia­zol-4(5*H*)-one with acetic anhydride. The presence of the acetyl and acet­oxy groups in the mol­ecule indicates that the starting thia­zole exists as a tautomer in the reaction mixture with exocyclic amino and enol moieties. The acetyl group is tilted slightly from the heterocyclic ring plane [dihedral angle = 4.46 (11)°], while the acet­oxy group is almost perpendicular to this ring [dihedral angle = 88.14 (12)°]. An intra­molecular acet­yl–meth­oxy C—H⋯O inter­action is noted. In the crystal, mol­ecules are connected into a three-dimensional architecture by C—H⋯O inter­actions.

## Related literature

For the biological activity of 2-aryl­amino­thia­zol-4-one derivatives, see: Chen *et al.* (2007 ▶); Eriksson *et al.* (2007 ▶); Lesyk & Zimenkovsky (2004 ▶); Lesyk *et al.* (2011 ▶); Ottana *et al.* (2005 ▶); Subtelna *et al.* (2010 ▶); Vassilev *et al.* (2006 ▶). For prototropic tautomerism studies, see: Subtelna *et al.* (2010 ▶); Lesyk *et al.* (2003 ▶); Vana *et al.* (2009 ▶).
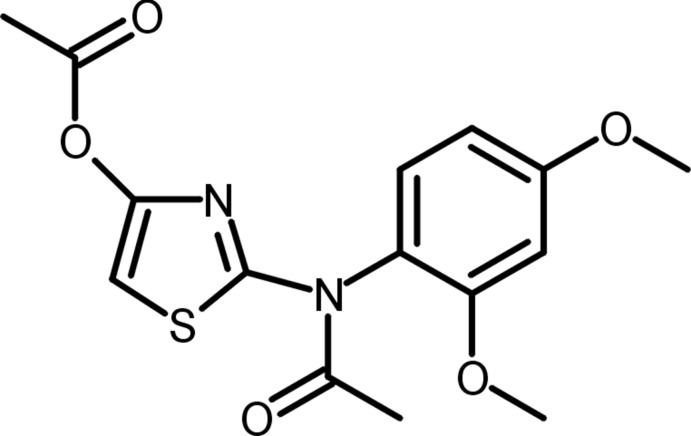


## Experimental

### 

#### Crystal data

C_15_H_16_N_2_O_5_S
*M* *_r_* = 336.36
Triclinic, 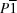

*a* = 9.1486 (11) Å
*b* = 9.3592 (13) Å
*c* = 10.2823 (8) Å
α = 69.212 (10)°
β = 82.910 (8)°
γ = 77.220 (11)°
*V* = 801.73 (16) Å^3^
*Z* = 2
Mo *K*α radiation
μ = 0.23 mm^−1^
*T* = 130 K
0.30 × 0.30 × 0.10 mm

#### Data collection

Agilent Xcalibur Atlas diffractometer
Absorption correction: multi-scan (*CrysAlis PRO*; Agilent, 2011 ▶) *T*_min_ = 0.919, *T*_max_ = 1.000
10576 measured reflections
3822 independent reflections
3281 reflections with *I* > 2σ(*I*)
*R*_int_ = 0.023

#### Refinement

*R*[*F* ^2^ > 2σ(*F* ^2^)] = 0.035
*wR*(*F* ^2^) = 0.093
*S* = 1.06
3822 reflections
212 parameters
H-atom parameters constrained
Δρ_max_ = 0.27 e Å^−3^
Δρ_min_ = −0.31 e Å^−3^

### 

Data collection: *CrysAlis PRO* (Agilent, 2011 ▶); cell refinement: *CrysAlis PRO*; data reduction: *CrysAlis PRO*; program(s) used to solve structure: *SHELXS97* (Sheldrick, 2008 ▶); program(s) used to refine structure: *SHELXL97* (Sheldrick, 2008 ▶); molecular graphics: *ORTEP-3 for Windows* (Farrugia, 2012 ▶); software used to prepare material for publication: *WinGX* (Farrugia, 2012 ▶) and *PLATON* (Spek, 2009 ▶).

## Supplementary Material

Crystal structure: contains datablock(s) I, global. DOI: 10.1107/S1600536813003474/tk5194sup1.cif

Structure factors: contains datablock(s) I. DOI: 10.1107/S1600536813003474/tk5194Isup2.hkl

Supplementary material file. DOI: 10.1107/S1600536813003474/tk5194Isup3.cml

Additional supplementary materials:  crystallographic information; 3D view; checkCIF report

## Figures and Tables

**Table 1:** Hydrogen-bond geometry (Å, °)

*D*—H⋯*A*	*D*—H	H⋯*A*	*D*⋯*A*	*D*—H⋯*A*
C9—H9*A*⋯O16	0.96	2.57	3.1838 (19)	122
C5—H5⋯O18^i^	0.93	2.47	3.2465 (18)	141
C15—H15⋯O8^ii^	0.93	2.52	3.3117 (18)	143
C17—H17*C*⋯O8^iii^	0.96	2.35	3.2945 (19)	170

Symmetry codes: (i) 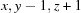; (ii) 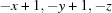; (iii) 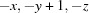.

**Fig. 1.**
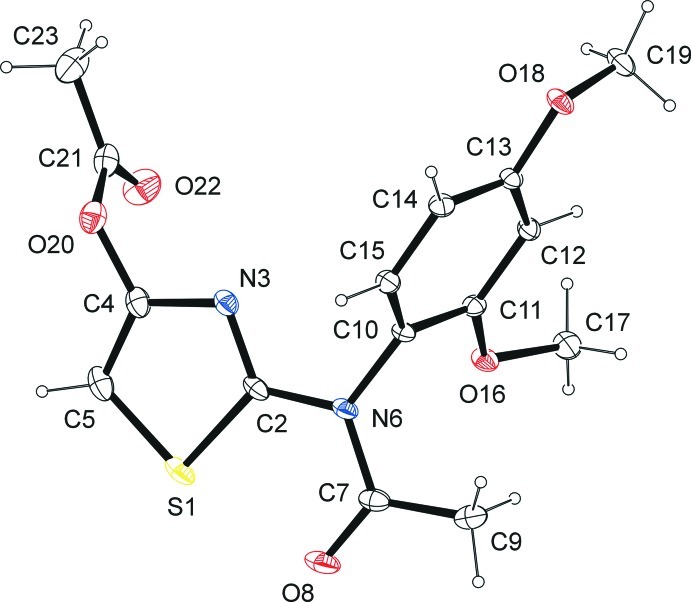
The molecular structure of (I) together with the atom-numbering scheme. Displacement ellipsoids are drawn at the 30% probability level and H atoms are shown as spheres of arbitrary radii.

**Fig. 2.**
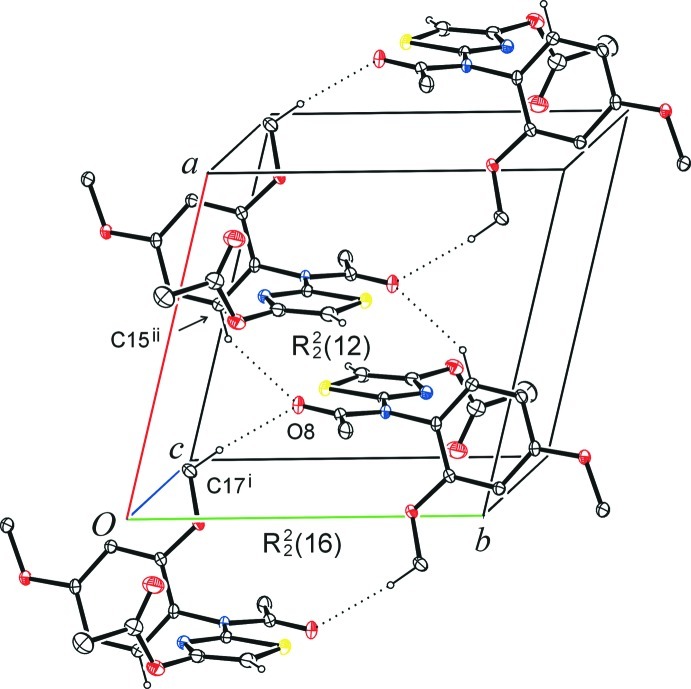
The hydrogen bonding (dotted lines) in (I), viewed approximately down the c axis. Symmetry codes: (i) -x, 1 - y, -z; (ii) 1 - x, 1 - y, -z. The ring graph symbols are shown. H atoms not involved in hydrogen bonds have been omitted for clarity.

**Fig. 3.**
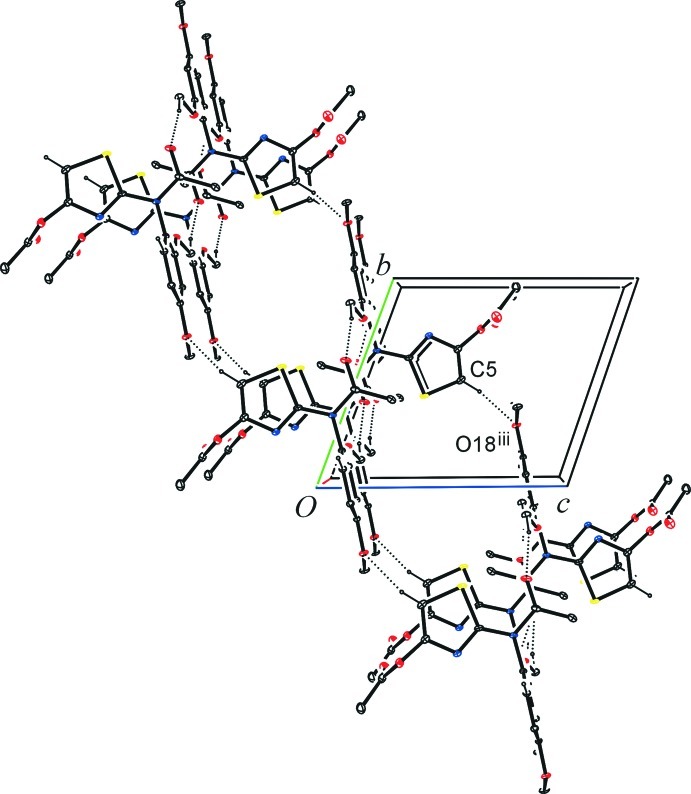
The hydrogen bonding (dotted lines) in (I), viewed approximately down the a axis. Symmetry code: (iii) x, -1 + y, 1 + z. H atoms not involved in hydrogen bonds have been omitted for clarity.

## supplementary crystallographic information

### Comment

2-Arylaminothiazol-4-one derivatives are of great importance in modern medicinal chemistry of anticancer agents (Lesyk & Zimenkovsky, 2004; Lesyk *et al.*, 2011). In particular, these heterocycles demonstrated inhibition of the HT29 cell line (colon cancer), characterized by a high COX-2 expression (Ottana *et al.*, 2005), as well as CDK1/cyclin B inhibition (Chen *et al.*, 2007). These effects were achieved by block of cell cycle progression at the G2/*M* phase border in reversible manner and induction of apoptosis (Vassilev *et al.*, 2006). Antagonizing stimulatory effects of free fatty acids at cell proliferation (inhibitory effect on tumor survival) in human breast cancer cell line (MDA-MB-231) was reported as well for the benzylidene-2-arylaminothiazol-4-ones (Eriksson *et al.*, 2007). Series of novel 5-arylidene-2-arylaminothiazol-4(5*H*)-ones were evaluated for the anticancer potential, *in vitro*, against the standard US National Cancer Institute's panel of 60 cancer cell lines. The majority of compounds showed significant cytotoxicity at micromolar and submicromolar concentrations; mean log(GI_50_) range -5.77 to -4.35. Some of the most potent compounds possessed selectively high effects on all leukemia cell lines at the submicromolar level (Subtelna *et al.*, 2010).

Prototropic tautomerism of 2-aminothiazol-4-ones presents an interesting target for studies of both molecular structures and spectroscopic properties (Subtelna *et al.*, 2010; Lesyk *et al.*, 2003; Vana *et al.*, 2009). Motivated by previous research of 2-arylaminothiazol-4-one derivatives, the aim of the present work was to synthesize the title compound (I) as a starting substance for further design of new anticancer agents.

The studies on the structure of compound (I), a product of the reaction of 2-(2,4-dimethoxyphenylamino)-1,3-thiazol-4-one with acetic anhydride, showed the presence of acetoxy and acetyl groups attached respectively at C4 and N6 positions of the compound (Fig. 1). This observation indicates that the starting material exists in the reaction mixture as a tautomer with an exocyclic amino nitrogen atom and additionally an enol moiety (*H—*)C5═C4—OH in the heterocyclic ring. The acetyl and acetoxy groups are oriented differently relative to the planar thiazole ring. The acetyl group at N6 is tilted only slightly [dihedral angle: 4.46 (11)°] whereas the acetoxy group at C4 is almost perpendicular to this ring [dihedral angle: 88.14 (12)°] (Fig. 1). Worthy of mention is the fact that the C7═ O8 and C21═O22 carbonyl groups are synperiplanar in relation to the C2—N6 and C4—O20, respectively. The torsion angles C2—N6—C7—O8 and C4—O20—C21—O22 are -1.16 (19) and 12.0 (2)°, respectively.

The methoxy groups on C11 and C14 of the phenyl ring are tilted to a small but statistically significant extent. The torsional angles C12—C11—O16—C17 and C12—C13—O18—C19 have the same value of 11.2 (2)°.

The phenyl ring of the 2,4-dimethoxyphenylamino substituent forms a dihedral angle of 73.17 (5)° with the planar thiazole ring. Such an orientation is supported by nonclassical C9—H9A···O16 intramolecular and O15—H15···O8^ii^ intermolecular hydrogen bonding (Table 1).

In the crystal lattice, the molecules of compound (I) are connected by three-centre hydrogen bonds C17^i^—H17C^i^···O8···H15^ii^—C15^ii^ into ribbons parallel to the *a* axis. According to the graph method for categorizing hydrogen bonds, this pattern can be classified as ring motifs *R*_2_^2^(16) and *R*_2_^2^(12) (Fig. 2). The neighbouring ribbons are linked further through the C5—H5···O18^iii^ contacts into layers growing parallel to the (011) plane (Fig. 3).

### Experimental

2-(2,4-Dimethoxyphenyl)thiazol-4-one (1 g) in the medium of acetic anhydride was refluxed for 2 h. The obtained solution was evaporated in vacuum and the residue was recrystallized twice from mixtures benzene–hexane (1:1) and CCl_4_ – hexane (1:1). Crystals were obtained from its methanol solution by evaporation at room temperature.

### Refinement

All H atoms were positioned into the idealized positions and were refined in the riding model approximation: C_methyl_—H = 0.96 Å, C(*sp*^2^)—H = 0.93 Å; *U*_iso_(H) = 1.2*U*_eq_(C) or 1.5*U*_eq_(C) for methyl H. The methyl groups were refined as rigid groups which were allowed to rotate.

### Crystal data

**Table:**

C_15_H_16_N_2_O_5_S	*Z* = 2
*M**_r_* = 336.36	*F*(000) = 352
Triclinic, *P*1	*D*_x_ = 1.393 Mg m^−^^3^
Hall symbol: -P 1	Melting point = 391–393 K
*a* = 9.1486 (11) Å	Mo *K*α radiation, λ = 0.71073 Å
*b* = 9.3592 (13) Å	Cell parameters from 4534 reflections
*c* = 10.2823 (8) Å	θ = 2.1–29.0°
α = 69.212 (10)°	µ = 0.23 mm^−^^1^
β = 82.910 (8)°	*T* = 130 K
γ = 77.220 (11)°	Plate, light-orange
*V* = 801.73 (16) Å^3^	0.30 × 0.30 × 0.10 mm

### Data collection

**Table:**

Agilent Xcalibur Atlas diffractometer	3822 independent reflections
Radiation source: fine-focus sealed tube	3281 reflections with *I* > 2σ(*I*)
Graphite monochromator	*R*_int_ = 0.023
Detector resolution: 10.3088 pixels mm^-1^	θ_max_ = 29.1°, θ_min_ = 2.1°
ω scans	*h* = −11→12
Absorption correction: multi-scan (*CrysAlis PRO*; Agilent, 2011)	*k* = −12→11
*T*_min_ = 0.919, *T*_max_ = 1.000	*l* = −13→13
10576 measured reflections	

### Refinement

**Table:**

Refinement on *F*^2^	Primary atom site location: structure-invariant direct methods
Least-squares matrix: full	Secondary atom site location: difference Fourier map
*R*[*F*^2^ > 2σ(*F*^2^)] = 0.035	Hydrogen site location: inferred from neighbouring sites
*wR*(*F*^2^) = 0.093	H-atom parameters constrained
*S* = 1.06	*w* = 1/[σ^2^(*F*_o_^2^) + (0.0432*P*)^2^ + 0.2365*P*] where *P* = (*F*_o_^2^ + 2*F*_c_^2^)/3
3822 reflections	(Δ/σ)_max_ < 0.001
212 parameters	Δρ_max_ = 0.27 e Å^−^^3^
0 restraints	Δρ_min_ = −0.31 e Å^−^^3^

### Special details

**Table:**

Geometry. All e.s.d.'s (except the e.s.d. in the dihedral angle between two l.s. planes) are estimated using the full covariance matrix. The cell e.s.d.'s are taken into account individually in the estimation of e.s.d.'s in distances, angles and torsion angles; correlations between e.s.d.'s in cell parameters are only used when they are defined by crystal symmetry. An approximate (isotropic) treatment of cell e.s.d.'s is used for estimating e.s.d.'s involving l.s. planes.
Refinement. Refinement of *F*^2^ against ALL reflections. The weighted *R*-factor *wR* and goodness of fit *S* are based on *F*^2^, conventional *R*-factors *R* are based on *F*, with *F* set to zero for negative *F*^2^. The threshold expression of *F*^2^ > σ(*F*^2^) is used only for calculating *R*-factors(gt) *etc*. and is not relevant to the choice of reflections for refinement. *R*-factors based on *F*^2^ are statistically about twice as large as those based on *F*, and *R*- factors based on ALL data will be even larger.

### Fractional atomic coordinates and isotropic or equivalent isotropic displacement parameters (Å^2^)

**Table:**

	*x*	*y*	*z*	*U*_iso_*/*U*_eq_	
S1	0.32458 (4)	0.43206 (4)	0.29160 (4)	0.02952 (11)	
C2	0.31474 (13)	0.61823 (14)	0.16952 (13)	0.0216 (3)	
N3	0.32110 (12)	0.72722 (12)	0.21915 (11)	0.0230 (2)	
C4	0.33664 (14)	0.66136 (16)	0.35971 (14)	0.0263 (3)	
C5	0.34049 (15)	0.50714 (17)	0.41887 (15)	0.0310 (3)	
H5	0.3502	0.4503	0.5132	0.037*	
N6	0.29963 (12)	0.65332 (12)	0.02702 (11)	0.0216 (2)	
C7	0.30067 (14)	0.53574 (15)	−0.02554 (15)	0.0260 (3)	
O8	0.31721 (12)	0.40055 (11)	0.05240 (12)	0.0346 (2)	
C9	0.28173 (17)	0.58190 (17)	−0.17857 (16)	0.0319 (3)	
H9A	0.1797	0.6325	−0.1990	0.048*	
H9B	0.3057	0.4908	−0.2054	0.048*	
H9C	0.3477	0.6521	−0.2293	0.048*	
C10	0.27699 (14)	0.81557 (14)	−0.05890 (12)	0.0194 (2)	
C11	0.13373 (14)	0.89309 (14)	−0.10249 (12)	0.0190 (2)	
C12	0.10876 (14)	1.05180 (14)	−0.18041 (12)	0.0195 (2)	
H12	0.0137	1.1044	−0.2108	0.023*	
C13	0.22900 (14)	1.13041 (14)	−0.21197 (12)	0.0197 (2)	
C14	0.37236 (14)	1.05301 (15)	−0.17013 (13)	0.0215 (3)	
H14	0.4517	1.1063	−0.1933	0.026*	
C15	0.39576 (14)	0.89529 (14)	−0.09341 (13)	0.0212 (3)	
H15	0.4914	0.8424	−0.0648	0.025*	
O16	0.02463 (10)	0.80501 (10)	−0.06499 (9)	0.0232 (2)	
C17	−0.11652 (15)	0.87342 (16)	−0.12963 (15)	0.0286 (3)	
H17A	−0.1002	0.9041	−0.2290	0.043*	
H17B	−0.1623	0.9632	−0.1043	0.043*	
H17C	−0.1814	0.7988	−0.0988	0.043*	
O18	0.21456 (10)	1.28646 (10)	−0.28607 (9)	0.0248 (2)	
C19	0.06590 (15)	1.37616 (15)	−0.31019 (16)	0.0302 (3)	
H19A	0.0173	1.3412	−0.3677	0.045*	
H19B	0.0707	1.4841	−0.3562	0.045*	
H19C	0.0099	1.3634	−0.2228	0.045*	
O20	0.35830 (11)	0.76068 (12)	0.42650 (10)	0.0312 (2)	
C21	0.23193 (17)	0.84306 (18)	0.47107 (14)	0.0322 (3)	
O22	0.11015 (12)	0.81482 (15)	0.47434 (13)	0.0452 (3)	
C23	0.2701 (2)	0.9684 (2)	0.51145 (18)	0.0450 (4)	
H23A	0.3330	0.9221	0.5896	0.067*	
H23B	0.1796	1.0297	0.5362	0.067*	
H23C	0.3223	1.0338	0.4344	0.067*	

### Atomic displacement parameters (Å^2^)

**Table:**

	*U*^11^	*U*^22^	*U*^33^	*U*^12^	*U*^13^	*U*^23^
S1	0.02693 (19)	0.01603 (17)	0.0364 (2)	−0.00475 (13)	−0.00359 (14)	0.00326 (14)
C2	0.0166 (6)	0.0141 (6)	0.0289 (6)	−0.0034 (4)	−0.0025 (5)	−0.0002 (5)
N3	0.0214 (5)	0.0197 (5)	0.0247 (5)	−0.0049 (4)	−0.0030 (4)	−0.0024 (4)
C4	0.0199 (6)	0.0288 (7)	0.0260 (6)	−0.0061 (5)	−0.0036 (5)	−0.0027 (5)
C5	0.0248 (7)	0.0315 (7)	0.0273 (7)	−0.0073 (6)	−0.0039 (5)	0.0033 (6)
N6	0.0226 (5)	0.0127 (5)	0.0274 (5)	−0.0042 (4)	−0.0026 (4)	−0.0034 (4)
C7	0.0198 (6)	0.0190 (6)	0.0405 (8)	−0.0052 (5)	0.0000 (5)	−0.0113 (6)
O8	0.0358 (6)	0.0157 (5)	0.0515 (7)	−0.0058 (4)	−0.0038 (5)	−0.0094 (4)
C9	0.0333 (8)	0.0275 (7)	0.0409 (8)	−0.0067 (6)	−0.0005 (6)	−0.0187 (6)
C10	0.0245 (6)	0.0127 (5)	0.0204 (6)	−0.0049 (5)	−0.0016 (5)	−0.0038 (5)
C11	0.0206 (6)	0.0179 (6)	0.0200 (6)	−0.0075 (5)	0.0002 (4)	−0.0062 (5)
C12	0.0198 (6)	0.0175 (6)	0.0200 (6)	−0.0041 (4)	−0.0019 (4)	−0.0044 (5)
C13	0.0261 (6)	0.0151 (6)	0.0174 (5)	−0.0064 (5)	0.0000 (5)	−0.0036 (5)
C14	0.0217 (6)	0.0196 (6)	0.0240 (6)	−0.0091 (5)	0.0004 (5)	−0.0059 (5)
C15	0.0196 (6)	0.0194 (6)	0.0243 (6)	−0.0038 (5)	−0.0023 (5)	−0.0065 (5)
O16	0.0211 (4)	0.0170 (4)	0.0303 (5)	−0.0082 (3)	−0.0020 (4)	−0.0035 (4)
C17	0.0240 (7)	0.0261 (7)	0.0361 (7)	−0.0109 (5)	−0.0056 (5)	−0.0061 (6)
O18	0.0244 (5)	0.0154 (4)	0.0291 (5)	−0.0067 (3)	−0.0030 (4)	0.0017 (4)
C19	0.0272 (7)	0.0177 (6)	0.0388 (8)	−0.0046 (5)	−0.0097 (6)	0.0015 (6)
O20	0.0287 (5)	0.0387 (6)	0.0271 (5)	−0.0107 (4)	−0.0029 (4)	−0.0092 (4)
C21	0.0339 (8)	0.0385 (8)	0.0226 (6)	−0.0085 (6)	−0.0043 (5)	−0.0066 (6)
O22	0.0312 (6)	0.0562 (8)	0.0564 (7)	−0.0090 (5)	−0.0007 (5)	−0.0290 (6)
C23	0.0506 (10)	0.0543 (11)	0.0388 (9)	−0.0167 (8)	−0.0028 (7)	−0.0222 (8)

### Geometric parameters (Å, º)

**Table:**

S1—C5	1.7240 (16)	C12—H12	0.9300
S1—C2	1.7394 (13)	C13—O18	1.3714 (14)
C2—N3	1.3067 (17)	C13—C14	1.3884 (18)
C2—N6	1.4008 (17)	C14—C15	1.3865 (17)
N3—C4	1.3669 (17)	C14—H14	0.9300
C4—C5	1.347 (2)	C15—H15	0.9300
C4—O20	1.3941 (17)	O16—C17	1.4364 (16)
C5—H5	0.9300	C17—H17A	0.9600
N6—C7	1.3855 (17)	C17—H17B	0.9600
N6—C10	1.4433 (15)	C17—H17C	0.9600
C7—O8	1.2212 (16)	O18—C19	1.4305 (16)
C7—C9	1.498 (2)	C19—H19A	0.9600
C9—H9A	0.9600	C19—H19B	0.9600
C9—H9B	0.9600	C19—H19C	0.9600
C9—H9C	0.9600	O20—C21	1.3663 (18)
C10—C11	1.3923 (17)	C21—O22	1.1945 (18)
C10—C15	1.3924 (17)	C21—C23	1.494 (2)
C11—O16	1.3667 (14)	C23—H23A	0.9600
C11—C12	1.3967 (17)	C23—H23B	0.9600
C12—C13	1.3989 (17)	C23—H23C	0.9600
			
C5—S1—C2	88.67 (7)	O18—C13—C12	123.00 (11)
N3—C2—N6	120.83 (11)	C14—C13—C12	121.30 (11)
N3—C2—S1	115.54 (10)	C15—C14—C13	119.16 (11)
N6—C2—S1	123.63 (10)	C15—C14—H14	120.4
C2—N3—C4	108.68 (11)	C13—C14—H14	120.4
C5—C4—N3	118.10 (13)	C14—C15—C10	120.40 (11)
C5—C4—O20	126.10 (12)	C14—C15—H15	119.8
N3—C4—O20	115.62 (12)	C10—C15—H15	119.8
C4—C5—S1	109.00 (10)	C11—O16—C17	117.33 (10)
C4—C5—H5	125.5	O16—C17—H17A	109.5
S1—C5—H5	125.5	O16—C17—H17B	109.5
C7—N6—C2	120.34 (11)	H17A—C17—H17B	109.5
C7—N6—C10	122.54 (11)	O16—C17—H17C	109.5
C2—N6—C10	117.07 (10)	H17A—C17—H17C	109.5
O8—C7—N6	119.83 (13)	H17B—C17—H17C	109.5
O8—C7—C9	122.69 (12)	C13—O18—C19	117.54 (10)
N6—C7—C9	117.48 (12)	O18—C19—H19A	109.5
C7—C9—H9A	109.5	O18—C19—H19B	109.5
C7—C9—H9B	109.5	H19A—C19—H19B	109.5
H9A—C9—H9B	109.5	O18—C19—H19C	109.5
C7—C9—H9C	109.5	H19A—C19—H19C	109.5
H9A—C9—H9C	109.5	H19B—C19—H19C	109.5
H9B—C9—H9C	109.5	C21—O20—C4	116.53 (11)
C11—C10—C15	120.31 (11)	O22—C21—O20	122.45 (14)
C11—C10—N6	119.22 (10)	O22—C21—C23	127.05 (15)
C15—C10—N6	120.42 (11)	O20—C21—C23	110.50 (13)
O16—C11—C10	116.17 (10)	C21—C23—H23A	109.5
O16—C11—C12	123.96 (11)	C21—C23—H23B	109.5
C10—C11—C12	119.87 (11)	H23A—C23—H23B	109.5
C11—C12—C13	118.95 (11)	C21—C23—H23C	109.5
C11—C12—H12	120.5	H23A—C23—H23C	109.5
C13—C12—H12	120.5	H23B—C23—H23C	109.5
O18—C13—C14	115.70 (10)		
			
C5—S1—C2—N3	−0.65 (10)	C15—C10—C11—O16	179.31 (11)
C5—S1—C2—N6	179.91 (11)	N6—C10—C11—O16	−3.12 (17)
N6—C2—N3—C4	−179.64 (11)	C15—C10—C11—C12	−0.44 (18)
S1—C2—N3—C4	0.91 (14)	N6—C10—C11—C12	177.13 (11)
C2—N3—C4—C5	−0.79 (17)	O16—C11—C12—C13	179.61 (11)
C2—N3—C4—O20	174.60 (11)	C10—C11—C12—C13	−0.66 (18)
N3—C4—C5—S1	0.31 (16)	C11—C12—C13—O18	−179.03 (11)
O20—C4—C5—S1	−174.54 (11)	C11—C12—C13—C14	1.49 (18)
C2—S1—C5—C4	0.17 (10)	O18—C13—C14—C15	179.30 (11)
N3—C2—N6—C7	176.71 (11)	C12—C13—C14—C15	−1.19 (19)
S1—C2—N6—C7	−3.88 (17)	C13—C14—C15—C10	0.06 (18)
N3—C2—N6—C10	−5.82 (17)	C11—C10—C15—C14	0.75 (18)
S1—C2—N6—C10	173.58 (9)	N6—C10—C15—C14	−176.78 (11)
C2—N6—C7—O8	−1.16 (19)	C10—C11—O16—C17	−168.59 (11)
C10—N6—C7—O8	−178.48 (11)	C12—C11—O16—C17	11.15 (17)
C2—N6—C7—C9	179.13 (11)	C14—C13—O18—C19	−169.30 (11)
C10—N6—C7—C9	1.80 (18)	C12—C13—O18—C19	11.20 (17)
C7—N6—C10—C11	74.41 (16)	C5—C4—O20—C21	−97.28 (16)
C2—N6—C10—C11	−102.99 (13)	N3—C4—O20—C21	87.75 (14)
C7—N6—C10—C15	−108.02 (14)	C4—O20—C21—O22	12.0 (2)
C2—N6—C10—C15	74.57 (15)	C4—O20—C21—C23	−167.56 (12)

### Hydrogen-bond geometry (Å, º)

**Table:**

*D*—H···*A*	*D*—H	H···*A*	*D*···*A*	*D*—H···*A*
C9—H9*A*···O16	0.96	2.57	3.1838 (19)	122
C5—H5···O18^i^	0.93	2.47	3.2465 (18)	141
C15—H15···O8^ii^	0.93	2.52	3.3117 (18)	143
C17—H17*C*···O8^iii^	0.96	2.35	3.2945 (19)	170

Symmetry codes: (i) *x*, *y*−1, *z*+1; (ii) −*x*+1, −*y*+1, −*z*; (iii) −*x*, −*y*+1, −*z*.
